# A Decade of Experience Between Open and Minimally Invasive Hepatectomies for Hepatocellular Carcinoma

**DOI:** 10.3390/medicina60111737

**Published:** 2024-10-23

**Authors:** Andrew Min-Gi Park, Ye In Christopher Kwon, Kush Savsani, Aadi Sharma, Yuzuru Sambommatsu, Daisuke Imai, Aamir Khan, Amit Sharma, Irfan Saeed, Vinay Kumaran, Adrian Cotterell, Marlon Levy, David Bruno, Seung Duk Lee

**Affiliations:** 1 School of Medicine, Virginia Commonwealth University, Richmond, VA 23298, USA; parkam5@vcu.edu (A.M.-G.P.); kwonyc@vcu.edu (Y.I.C.K.); kush.savsani1@vcuhealth.org (K.S.); asharma8@vcu.edu (A.S.); 2 Department of Transplant Surgery, Virginia Commonwealth University Health System, West Hospital, 1200 E. Broad St, Richmond, VA 23298, USA; yuzuru.sambommatsu@vcuhealth.org (Y.S.); daisuke.imai@vcuhealth.org (D.I.); aamir.khan@vcuhealth.org (A.K.); amit.sharma@vcuhealth.org (A.S.); muhammad.saeed@vcuhealth.org (I.S.); vinay.kumaran@vcuhealth.org (V.K.); adrian.cotterell@vcuhealth.org (A.C.); marlon.levy@vcuhealth.org (M.L.); david.bruno@vcuhealth.org (D.B.)

**Keywords:** hepatocellular carcinoma, hepatectomy, laparoscopy, robotic surgical procedures

## Abstract

*Background and Objectives*: Hepatic resection offers promising outcomes for patients with hepatocellular carcinoma (HCC) but can be constrained by factors like patient suitability. Continuous advancements in laparoscopic and robotic technologies have made minimally invasive hepatectomies (MIHs) a viable alternative to open hepatectomies with benefits in terms of recovery and complications. *Materials and Methods*: We completed a retrospective review on 138 HCC patients who underwent OH or MIH between 2010 and 2020 at the Hume-Lee Transplant Center. Univariate and multivariate analyses were completed on demographic, clinical, and tumor-specific data to assess the impact of these variables on overall and disease-free survival at 1, 3, and 5 years. Preoperative metrics like length of hospital stay (LOS) and operation duration were also evaluated. *Results*: Of the 109 OH and 29 MIH patients, MIH patients demonstrated shorter LOS and operative times. However, overall survival (OS) and disease-free survival (DFS) were similar between groups, with no significant variations in 1-, 3-, and 5-year survival rates. Age > 60 years and a lack of preoperative transcatheter arterial chemoembolization (TACE) were significant predictors of inferior OS and DFS in multivariate analyses. *Conclusions*: MIH is an efficient substitute for OH with comparable survival, even in older patients. The reduced LOS and operation time enhance its feasibility, and older patients previously denied for curative resection may qualify for MIH. Preoperative TACE also enhances survival outcomes, emphasizing its general role in managing resectable HCCs. Both robotic and laparoscopic hepatectomies offer acceptable short- and long-term clinical outcomes, highlighting MIH as the standard choice for HCC patients.

## 1. Introduction

Hepatocellular carcinoma (HCC) is the third leading cause of cancer death worldwide, accounting for 70–85% of all primary liver cancers [1]. HCC is typically associated with hepatitis B or C, alcohol use, and non-alcoholic fatty liver disease [2]. With a 5-year survival rate of approximately 18%, its prognosis remains poor without treatment. The World Health Organization estimates more than 1 million cancer deaths from liver cancer by 2030 [2,3]. The stage of disease primarily determines treatment options for HCC. Surgical treatment offers the highest cure rates but is reserved for early-stage (I/II) HCC with minimal liver disease [2,4,5,6]. Liver transplantation, while preferred for HCC patients with cirrhosis (80% of HCC cases), is limited by organ shortage and strict selection criteria [7,8]. Thus, the role of surgical resection in patients with localized HCC has garnered greater interest, along with improvements in clinical decision algorithms [9,10]. Previously, surgical resection was reserved for patients with a solitary tumor of any size or up to three nodules of <3 cm [11,12]. However, technological advancements in techniques and devices have allowed major hepatectomies to be performed in patients with larger, more malignant liver lesions [13]. Laparoscopic techniques have long been established as a safe and effective alternative to open surgery, providing improved visualization, magnification, less intraoperative blood loss, and more meticulous dissection [14,15,16]. Smaller incisions in the anterior abdominal wall minimize interruption in portosystemic collateral vessels, reducing liver failure and ascites recurrence in patients with severe cirrhosis [17,18,19,20].

Furthermore, robotic platforms have addressed constraints associated with laparoscopic hepatectomies [21]. Robotic systems can achieve safer surgical margins by adhering to curvilinear resection planes through well-articulated instruments and contact ultrasonography via robotic probes [22,23]. Improved visualization, ergonomics, and range of motion within the abdominal cavity may enhance handling, dexterity, and fine dissection maneuvers [24]. The present study evaluates the short- and long-term outcomes of patients receiving open hepatectomy surgery (OH) versus minimally invasive hepatectomy surgery (MIH) for hepatocellular carcinoma. Through a decade of experience at a tertiary referral center, this study aims to assess the effectiveness and safety of MIH in managing HCC, potentially influencing surgical protocols and patient care.

## 2. Materials and Methods

Patient confidentiality and privacy were strictly maintained, and all data were anonymized to protect the participants’ identities. The Institutional Review Board at Virginia Commonwealth University approved our study under protocol number HM20007405 in 2021.

### 2.1. Patient Selection

This single-center, retrospective study evaluated the medical records of 134 HCC patients who underwent liver resections at the Hume-Lee Transplant Center in Richmond, Virginia, from 1 January 2010 to 31 December 2020. Patients were stratified into OH and MIH groups. Patients with American Joint Committee on Cancer (AJCC) stages I–IV diagnoses who underwent curative-intent liver resection were included, as well as those who had undergone preoperative treatment including TACE, RFA, resection, or a combination of treatments.

We collected comprehensive demographics and clinical data, including tumor-specific characteristics like the number, total and largest size, AJCC three-tier grading, microvascular and macrovascular invasion, and lymphatic and capsular invasion. Preoperative labs and morbidity-related data on the length of hospital stay, operative duration, transfusion, and estimated resection margins were reported. Our primary outcomes were overall survival at 1-, 3-, and 5-year intervals, defined as the time interval between the liver resection and death or the most recent follow-up. Our secondary outcomes were disease-free survival at these time intervals, defined as the period after hepatectomy without signs of HCC recurrence.

### 2.2. Statistical Analysis

Categorical variables were reported as percentages, and continuous variables as means with standard deviations (SDs). Surgical factors like the resection margin and technique were also evaluated. For comparing patients undergoing OH or MIH, Pearson’s Chi-square test or Fisher’s exact test were used for categorical variables and the Kruskal–Wallis rank-sum test was used for continuous variables. Kaplan–Meier plots were used to analyze overall and disease-free survival at 1-, 3-, and 5-year intervals. Cox proportional hazards regression analyzed predictors of overall survival and of disease-free survival, with significant variables (age and prior TACE) from univariate analysis included in the multivariate model. Hazard ratios (HRs) and 95% confidence intervals (CIs) were used to determine significance for predicting overall survival. Statistical analyses were conducted using Python (Ver. 3.8.18), where *p*-values were based on two-sided statistical tests with *p* < 0.05 considered significant.

## 3. Results

### 3.1. Patient Characteristics

Between 2010 and 2020, 109 patients diagnosed with HCC received OH while 29 received MIH (Table 1). Within the MIH cohort, 15 patients underwent robotic surgery (2016–2020). Fourteen patients underwent conventional laparoscopic surgery (only two cases were performed throughout 2018 and 2019). There was no significant difference between the MIH and OH groups in terms of gender (82.8% vs. 66.1%; *p* = 0.112) or racial distribution (*p* = 0.070). However, the mean age at the time of surgery was significantly higher in the MIH group than the OH group (66.9 years vs. 60.1 years; *p* = 0.015).

Both groups had similar rates of cirrhosis, prior cancers, and preoperative therapy (*p* = 0.233; *p* = 0.767; *p* = 0.732). However, a higher proportion of the MIH group had three or fewer tumors than the OH group (96.6% vs. 80.7%; *p* = 0.045). While the largest tumor sizes found were similar, the MIH group showed a smaller total tumor size (4.9 cm vs. 7.3 cm; *p* = 0.031). There were no significant differences in differentiation grading, microvascular or macrovascular invasion, lymphatic or capsular invasion, or AJCC staging.

### 3.2. Clinical Outcomes

Univariate analysis showed that age > 60 was associated with worse overall survival (OS) (HR 1.891, CI: 1.156–3.093; *p* = 0.011) (Table 2). Multivariate analysis confirmed the significance between age and overall survival (HR 1.848, CI: 1.122–3.046; *p* = 0.016). A lack of preoperative TACE showed improved overall survival in univariate analyses (HR 0.393, CI: 0.221–0.698; PI = 0.001) and multivariate analyses (HR 0.398, CI: 0.222–0.712; *p* = 0.002).

Age ≥ 60 years and the absence of preoperative TACE showed significant association with disease-free survival (DFS) as well (Table 3). Age > 60 years worsened disease-free survival in both univariate (HR 1.918, CI: 1.076–3.418; *p* = 0.027) and multivariate analyses (HR 1.982, CI: 1.169–3.359; *p* = 0.024). A lack of preoperative TACE was associated with improved DFS in univariate analysis (HR 0.360, CI: 0.178–0.727; *p* = 0.004).

**Figure 1:**
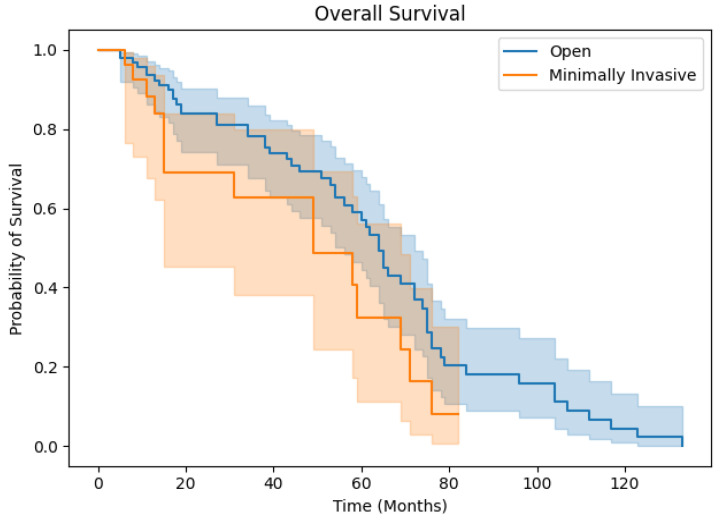
Overall survival for OH and MIH patients.

**Figure 2:**
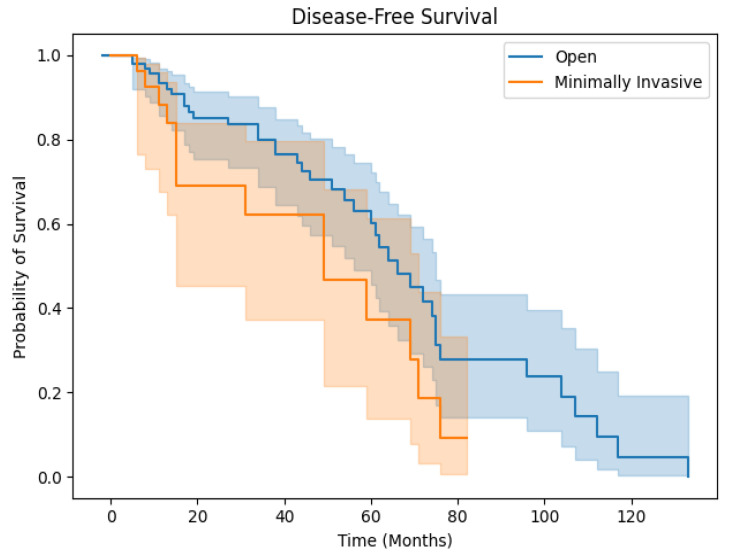
Disease-free survival for OH and MIH patients.

Surgical technique did not impact OS (*p* = 0.484) between OH and MIH patients. For MIH patients, the 1-, 3-, and 5-year OS rates were 79.7%, 52.9%, and 44.1%, while the 1-, 3-, and 5-year OS rates for OH patients were 79.3%, 64.2%, and 47.9% (Figure 1). The disease-free survival rates were comparable as well (*p* = 0.729). For MIH patients, the 1-, 3-, and 5-year DFS rates were 73.2%, 53.4%, and 40.0%, while the 1-, 3-, and 5-year OS rates for OH patients were 70.5%, 48.3%, and 40.0% (Figure 2). No MIH patients survived beyond 6.67 years.

Patients undergoing MIH had shorter LOS than those in the open surgery group (5.4 days vs. 8.8 days; *p* = 0.003) and shorter operation times (261.0 min vs. 338.8 min; *p* = 0.004 (Table 4). There were no considerable differences in intraoperative blood transfusion volume between MIH and OH patients (700.0 mL vs. 1161.6 mL; *p* = 0.263) or resection margins (negative margins < 10 mm: 11 vs. 55; >10 mm: 11 vs. 30; positive margins: 3 vs. 16; *p* = 0.392).

## 4. Discussion

Hepatic resection for HCC remains challenging for patients and surgeons, but laparoscopic approaches have remained popular for over three decades thanks to advancements in surgical techniques and improved equipment [25]. Several studies have illustrated the benefits of laparoscopic approaches among HCC patients, including reduced hospitalization, blood loss, and postoperative morbidity while maintaining comparable oncological results to open resection [14,26,27,28]. With the recent emergence of robot-assisted liver resections, the safety and feasibility of MIH are subject to continual assessment. Our analysis represents a decade of experience with conventional and robot-assisted laparoscopic liver resections at a high-volume, tertiary referral center, in comparison to traditional open procedures.

OH and MIH patients were similar in terms of demographics, comorbidities, and tumor characteristics, with the notable exception of age. Despite no definitive consensus regarding the outcomes of OH and MIH among higher-risk elderly patients, it is crucial to identify patients who may benefit most from minimally invasive alternatives. A recent meta-analysis indicates that elderly patients are more prone to postoperative morbidity and short-term mortality with laparoscopic hepatectomies [29]. The associated risk rises as individuals age, peaking when patients reach their seventies, before stabilizing in septuagenarians [29]. However, our data demonstrate that patients undergoing MIH (robotic and laparoscopic) may experience similar survival benefits compared to those undergoing OH, despite the age discrepancy.

This supports studies showing MIH’s safety and feasibility profiles among elderly patients, possibly attributed to the significantly decreased length of hospital stay among MIH patients [30,31]. Elderly patients who spend a longer time in the Intensive Care Unit (ICU) may develop disabilities that impact overall quality of life, with national analysis reporting a median LOS of 3 days among MIH recipients compared to 6 days among OH patients [14,32]. Other centers reported a slightly higher LOS of 7 days for MIH patients, but still lower than the 9 days seen in OH patients [33]. Our data regarding LOS are within these reported ranges. Additionally, postoperative infections are among the most frequent complications in elderly patients. While not addressed in this study, it is plausible that reducing severe postoperative wound infections and hastening the recovery process may have contributed to similar complication rates in both groups [14].

As the screening apparatus for liver cirrhosis continues to improve globally, elderly patients face higher risks of complications following liver resection and HCC [34]. Our experience indicates that sexagenarians had increased rates of mortality. Despite our patients being mostly sexagenarians, there were no differences in the presence of cirrhosis between OH and MIH groups. The benefits of the minimally invasive approach may decrease in patients aged 80 and above [35]. Nevertheless, this has not been demonstrated among our patients undergoing MIH. Liver transplantation is another important alternative to resection in HCC patients with cirrhosis, but elderly patients nearing their 70s are mostly excluded due to comorbidities [36]. Our data demonstrate that elderly patients previously denied an opportunity for a potentially curative liver resection can be offered the viable and potentially favorable treatment option of MIH.

The differences in operative time between OH and MIH are notable, as a longer operative time is a significant predictor of surgical morbidity after laparoscopic liver resections [37]. Unlike recent national and institutional retrospective analyses showing no difference in operative times, our experience demonstrates a significantly reduced duration of operation in favor of MIH [14,33]. This may be due to MIH patients having lower tumor numbers and sizes. Indeed, minor resections have decreased operative times in laparoscopic liver resections and may be associated with lower blood loss and intraoperative transfusion rates [38]. While our study showed no significant difference in intraoperative transfusion rates, MIH patients tended to require less overall transfusion volume.

A prior history of TACE was associated with lower mortality in patients undergoing both OH and MIH. Preoperative TACE identifies latent intrahepatic metastatic foci, improves the resectability of HCCs by reducing tumors that are initially borderline resectable or unresectable, allows adequate time for therapy when liver function is compromised, and enhances the overall survival and disease-free survival rates following curative resection [39]. Multiple retrospective studies have demonstrated its effectiveness in patients with intermediate-stage HCC who benefit from superior oncological results with OH or MIH [40]. These patients tend to have large and multifocal HCC without intrahepatic macrovascular invasion or extrahepatic metastases [41]. This is a salient point for patients undergoing OH who have larger and more numerous tumors compared to MIH patients, albeit a statistically insignificant difference. Certain clinicians may not advocate for preoperative TACE in patients with resectable HCC being evaluated for surgical intervention. One of the key factors to consider is that TACE may affect well-differentiated HCC while incompletely eradicating poorly differentiated cells [42]. Consequently, preoperative TACE may increase the chance of HCC metastasis via the portal venous system. However, our experience shows selective chemoembolization of HCC tumors prior to hepatectomy may confer additional survival benefits for HCC patients.

Furthermore, it is known that MIH has a steep learning curve that requires concomitant training in both laparoscopic and robotic techniques [43]. Despite increasing variation in operator experience as MIH evolves, MIH offers several key advantages compared to OH. Unlike OH, MIH may minimize the damage to adherent structures and the liver through manipulation only inside the subphrenic rib cage. Laparoscopic instruments can directly access the space caudally with minimal damage to the cage and minimal mobilization or compression of the liver [44]. Second, MIH may have advantages for the treatment of intrahepatic recurrence compared to OH. While our short- and long-term data showed non-inferior DFS of MIH compared to OH, it is likely that reoperation on the liver is made more difficult once adhesions are formed. Notably, MIH may be associated with lower rates of adhesion formation, allowing for better visibility and maneuverability even in the small surgical fields between adhesions [44]. Comparable OS and DFS after MIH may be due to reduced functional deterioration of the liver [25]. With reduced adhesions, the process of repeat treatments becomes more accessible, decreasing mortality among patients with liver insufficiency.

MIH has its disadvantages, as maintaining orientation can be difficult due to the lack of fine perceptible sensation and visibility of the entire surgical field. However, effective simulation of minor anatomical resections can ensure precise localization of the tumor within the resected area and adequate surgical margins. This may result in reduced postoperative complications, diminished residual ischemic/congestive parenchyma, and potentially lower recurrence risk [45]. Robotic technology also confers numerous benefits compared to conventional laparoscopic techniques [46]. Features such as Endowrist technology provide a remarkable seven degrees of freedom in hand manipulation. Additionally, robotic systems enable operating surgeons to access lesions located in the posterior superior region, enhance their suturing, reduce physiological tremors, and promote better ergonomics [46]. Overall, these advancements in MIH have likely reduced operative time and other morbidities at our institution, and we resultantly demonstrate acceptable short- and long-term clinical results with both robotic and laparoscopic techniques.

### Limitations

While our study provides valuable insight into the outcomes and survival rates of patients who have undergone OH and MIH for HCC, there are several limitations. First, this was a single-center analysis, which limits the generalizability of the findings. Second, the retrospective nature of our study introduces a certain level of selection bias, as the choice between OH and MIH was determined by physicians based on clinical judgment and patient characteristics. Third, the lack of statistical significance in certain variables may be due to the insufficient power to identify a potential association. Fourth, another limitation of our study includes the unequal distribution of sample sizes between the OH group and MIH group, with only 29 MIH patients (including 15 robotic-assisted cases) in contrast to the 109 patients in the OH group. Such variation potentially limits the generalizability of our findings and underscores the need for more equitably matched groups in future studies to better evaluate the differences between these surgical approaches. Finally, this study did not account for adjuvant therapies. Future studies with larger, multi-center cohorts and prospective design are needed for validating our findings and further exploring the benefits and limitations of MIH in the treatment of HCC, especially in the evaluation of postoperative complications to better detail the safety profiles of each surgical technique. More studies are also needed to compare clinical outcomes between laparoscopic surgeries and robotic surgeries using large sets of single-center or multi-center clinical data. Further research on the implications of robotic surgeries specifically on perioperative management and outcomes in the scope of hepatectomies on patients with HCC will be needed as they become more commonplace due to improving learning curves. Cost analysis studies are also needed to determine the financial impact of robotic and laparoscopic resections on optimizing resource allocation and decision-making.

## 5. Conclusions

This paper represents a recent decade of experience with both OH and MIH at a tertiary referral center. For patients with HCC, MIH—both robot-assisted and conventional laparoscopic—may confer faster recovery and reduced operative times compared to OH. Selected patients may also receive survival benefits when preoperatively treated with TACE. Despite significantly increased age among patients undergoing MIH, these procedures showed comparable short- and long-term oncologic outcomes when compared to OH.

## Figures and Tables

**Table 1:** Baseline characteristics of patients undergoing open or minimally invasive hepatectomies.

Characteristics	Open (*n* = 109)	Minimally Invasive (*n* = 29)	*p*-Value
Sex, *n* (%)			0.112
Male	72 (66.1)	24 (82.8)	
Female	37 (33.9)	5 (17.2)	
Age (year), mean (SD)	60.1 (13.4)	66.9 (12.1)	0.015
Race, *n* (%)			0.070
White	46 (42.2)	14 (48.3)	
Black	49 (45.0)	11 (37.9)	
Asian	14 (12.8)	2 (6.9)	
Hispanic	0 (0)	1 (3.4)	
Other	0 (0)	1 (3.4)	
WBC (×10^9^/L), mean (SD)	6.8 (2.4)	5.8 (2.0)	0.059
PLT (×10^9^/L), *n* (%)			0.828
<225	72 (66.1)	20 (69.0)	
≥225	37 (33.9)	9 (31.0)	
ALT (U/L), *n* (%)			0.648
<80	76 (70.4)	22 (75.9)	
≥80	32 (29.6)	7 (24.1)	
Serum sodium (mmol/L), mean (SD)	139.0 (3.1)	140.0 (2.6)	0.125
Serum creatinine (μmol/L), mean (SD)	0.9 (0.3)	1.0 (0.5)	0.042
TBIL (μmol/L), mean (SD)	0.8 (0.7)	0.8 (0.5)	0.750
INR, mean (SD)	1.1 (0.3)	1.1 (0.1)	0.511
AFP (ng/mL), mean (SD)			0.039
<200	76 (74.5)	27 (93.1)	
≥200	26 (25.5)	2 (6.9)	
MELD-XI, mean (SD)	8.7 (3.5)	8.8 (2.8)	0.852
Cirrhosis, *n* (%)	96 (88.1)	23 (79.3)	0.233
Prior cancer, *n* (%)	15 (13.8)	5 (17.4)	0.767
Preoperative therapy, *n* (%)			0.732
No therapy	79 (73.1)	24 (82.8)	
TACE only	19 (17.6)	2 (6.9)	
RFA only	3 (2.8)	1 (3.4)	
Radiation only	1 (0.9)	0 (0)	
Resection only	1 (0.9)	0 (0)	
Combination	5 (4.6)	2 (6.9)	
Tumor number			0.045
≤3	88 (80.7)	28 (96.6)	
>3	21 (19.3)	1 (3.4)	
Largest tumor size (cm)	5.6 (4.3)	4.3 (3.3)	0.138
Total tumor size (cm)	7.3 (5.2)	4.9 (4.1)	0.031
Tumor differentiation, *n* (%)			0.372
G1	11 (10.7)	5 (19.2)	
G2	62 (60.2)	16 (61.5)	
G3	30 (29.1)	5 (19.2)	
Microvascular invasion, *n* (%)	59 (54.1)	10 (34.5)	0.118
Macrovascular invasion, *n* (%)	29 (26.6)	5 (17.2)	0.460
Lymphatic invasion, *n* (%)	5 (4.6)	1 (3.4)	1.000
Capsular invasion, *n* (%)	69 (63.3)	18 (62.1)	0.643
AJCC staging, *n* (%)			0.159
I–II	78 (76.5)	22 (91.7)	
III–IV	24 (23.5)	2 (8.3)	

Abbreviations: AFP: alpha fetoprotein; ALT: alanine aminotransferase; AJCC: American Joint Committee on Cancer; INR: international normalized ratio; MELD-XI: model for end-stage liver disease excluding INR; PLT: platelet; RFA: radiofrequency ablation; SD: standard deviation; TACE: transcatheter arterial chemoembolization; TBIL: total bilirubin; WBC: white blood count.

**Table 2:** Cox regression analysis of predictors of overall survival.

	Univariate			Multivariate		
	HR	95% CI	*p*-Value	HR	95% CI	*p*-Value
Sex (ref: female)			0.602			
Male	1.137	0.701–1.845				
Age (ref: <60)			0.011			0.016
≥60	1.891	1.156–3.093		1.848	1.122–3.046	
WBC (ref: <3.5)			0.350			
≥3.5	0.615	0.222–1.704				
PLT (ref: <225)			0.778			
≥225	0.933	0.577–1.510				
ALT (ref: <80)			0.634			
≥80	0.870	0.492–1.541				
TBIL (ref: <2)			0.398			
≥2	1.663	0.512–5.402				
AFP (ref: <400)			0.695			
≥400	0.874	0.447–1.711				
MELD-XI (ref: <15)			0.710			
≥15	1.250	0.385–4.051				
Cirrhosis			0.979			
Present	1.008	0.565–1.799				
Prior TACE			0.001			0.002
No	0.393	0.221–0.698		0.398	0.222–0.712	
Prior RFA			0.077			
No	0.276	0.066–1.150				
Prior resection			0.291			
No	2.147	0.520–8.858				
Resection margin (ref: positive)						
<10 mm	1.756	0.924–3.339	0.086			
>10 mm	1.204	0.610–2.379	0.593			
Surgical technique (ref: open)			0.072			
Lap/Robotic	1.703	0.953–3.044				
Tumor number (ref: <3)			0.715			
>3	1.174	0.496–2.779				
Mean largest tumor size (ref: <5)			0.135			
≥5	0.678	0.408–1.128				
Mean tumor size (ref: <10)			0.471			
≥10	0.781	0.399–1.529				
Microvascular invasion			0.315			
Present	0.786	0.492–1.257				
Macrovascular invasion			0.349			
Present	0.744	0.400–1.382				
Lymphatic invasion			0.731			
Present	0.706	0.097–5.142				
Ductal invasion			0.714			
Present	0.870	0.411–1.838				
AJCC staging (ref: I–II)			0.307			
II–IV	0.680	0.324–1.424				

Abbreviations: AFP: alpha fetoprotein; ALT: alanine aminotransferase; AJCC: American Joint Committee on Cancer; CI: confidence interval; HR: hazard ratio; INR: international normalized ratio; MELD-XI: model for end-stage liver disease excluding INR; PLT: platelet; RFA: radiofrequency ablation; SD: standard deviation; TACE: transcatheter arterial chemoembolization; TBIL: total bilirubin; WBC: white blood count.

**Table 3:** Cox regression analysis of predictors of disease-free survival.

	Univariate			Multivariate		
	HR	95% CI	*p*-Value	HR	95% CI	*p*-Value
Sex (ref: female)			0.635			
Male	1.150	0.646–2.049				
Age (ref: <60)			0.027			0.024
≥60	1.918	1.076–3.418		1.982	0.169–0.712	
WBC (ref: <3.5)			0.302			
≥3.5	0.533	0.162–1.759				
PLT (ref: <225)			0.643			
≥225	0.876	0.500–1.534				
ALT (ref: <80)			0.606			
≥80	0.834	0.418–1.663				
TBIL (ref: <2)			0.514			
≥2	1.613	0.383–6.792				
AFP (ref: <400)			0.547			
≥400	0.792	0.370–1.693				
MELD-XI (ref: <15)			0.745			
≥15	0.719	0.098–5.263				
Cirrhosis			0.883			
Present	1.052	0.536–2.063				
Prior TACE			0.004			0.004
No	0.360	0.178–0.727		0.347	0.169–0.712	
Prior RFA			0.130			
No	0.216	0.030–1.572				
Prior resection			0.269			
No	2.234	0.536–9.306				
Resection margin (ref: positive)						
<10 mm	1.928	0.908–4.093	0.087			
>10 mm	1.135	0.485–2.657	0.770			
Surgical technique (ref: open)			0.060			
Lap/Robotic	1.815	0.975–3.379				
Tumor number (ref: <3)			0.893			
>3	0.931	0.330–2.631				
Mean largest tumor size (ref: <5)			0.098			
≥5	0.589	0.315–1.103				
Mean tumor size (ref: <10)			0.310			
≥10	0.620	0.246–1.562				
Microvascular invasion			0.405			
Present	0.794	0.461–1.366				
Macrovascular invasion			0.590			
Present	0.824	0.407–1.667				
Lymphatic invasion			0.756			
Present	0.730	0.100–5.335				
Ductal invasion			0.728			
Present	0.858	0.362–2.033				
AJCC staging (ref: I–II)			0.181			
II–IV	0.497	0.179–1.383				

Abbreviations: AFP: alpha fetoprotein; ALT: alanine aminotransferase; AJCC: American Joint Committee on Cancer; CI: confidence interval; HR: hazard ratio; INR: international normalized ratio; MELD-XI: model for end-stage liver disease excluding INR; PLT: platelet; RFA: radiofrequency ablation; SD: standard deviation; TACE: transcatheter arterial chemoembolization; TBIL: total bilirubin; WBC: white blood count.

**Table 4:** Comparisons of clinical outcomes between open and minimally invasive hepatectomies.

	Open (n = 109)	Minimally Invasive (n = 29)	*p*-Value
LOS (days), mean (STD)	8.8 (5.7)	5.4 (2.9)	0.003
Operation time (minutes), mean (STD)	338.8 (129.1)	261.0 (101.2)	0.004
Transfusion			0.317
Yes	25	4	
No	82	25	
Transfusion amount, median (IQR)	1161.6 (788.4)	700.0 (285.8)	0.263
Resection margin			0.392
Negative <10 mm, n (%)	55 (54.5)	11 (44.0)	
Negative >10 mm, n (%)	30 (29.7)	11 (44.0)	
Positive, n (%)	16 (15.8)	3 (12.0)	

Abbreviations: IQR: interquartile range; LOS: length of stay; STD: standard deviation.

## Data Availability

Research data are not shared.
